# Cervical Artery Dissection and Sports

**DOI:** 10.3389/fneur.2021.663830

**Published:** 2021-05-31

**Authors:** Stefan T. Engelter, Christopher Traenka, Caspar Grond-Ginsbach, Tobias Brandt, Maani Hakimi, Bradford B. Worrall, Stephanie Debette, Alessandro Pezzini, Didier Leys, Turgut Tatlisumak, Christian H. Nolte, Philippe Lyrer

**Affiliations:** ^1^Department of Neurology and Stroke Center, University Hospital Basel and University of Basel, Basel, Switzerland; ^2^Neurology and Neurorehabilitation, University Department of Geriatric Medicine FELIX PLATTER, University of Basel, Basel, Switzerland; ^3^Department of Vascular and Endovascular Surgery, Heidelberg University Hospital, Heidelberg, Germany; ^4^Schweizerische Unfallversicherungsanstalt (SUVA), Swiss National Accident Insurance Institution, Lucerne, Switzerland; ^5^Department of Vascular Surgery, Luzerner Kantonsspital, Lucerne, Switzerland; ^6^Departments of Neurology and Public Health Sciences, University of Virginia Health System, Charlottesville, VA, United States; ^7^Department of Neurology, Bordeaux University Hospital, Bordeaux, France; ^8^Inserm U1219, Bordeaux, France; ^9^Department of Clinical and Experimental Sciences, Neurology Clinic, University of Brescia, Brescia, Italy; ^10^Univ-Lille, Inserm U1171, Centre Hospitalier Universitaire (CHU) Lille, Lille, France; ^11^Department of Neurology, Sahlgrenska University Hospital and Department of Clinical Neuroscience, Institute for Neuroscience and Physiology, Sahlgrenska Academy at University of Gothenburg, Gothenburg, Sweden; ^12^Department of Neurology, and Center for Stroke Research Berlin Charité–Universitätsmedizin Berlin, Berlin, Germany

**Keywords:** cervical artery dissection, carotid artery dissection, vertebral artery dissection, sport, physical acitivity

## Abstract

Cervical artery dissection (CeAD) occurring in the context of sports is a matter of concern for CeAD patients. They seek advice on the role of sports in CeAD and on the safety of resuming sports after CeAD. The scarcity of studies and guidelines addressing these issues poses a challenge. We aimed at summarizing the current knowledge about CeAD and sports in order to provide an informed, comprehensive opinion for counseling CeAD patients. We took into account pathophysiological considerations, observations of cases reports, series, and registries, and conclusions by analogy from aortic dissection or inherited connective tissue syndromes. In summary, practicing active sports as the cause of CeAD seems uncommon. It seems recommendable to refrain from any kind of sports activities for at least 1 month, which can be extended in case of an unfavorable clinical or neurovascular course. We recommend starting with sport activities at low intensity—preferably with types of endurance sports—and to gradually increase the pace in an individually tailored manner, taking into circumstances of the occurrences of the CeAD in the individual patient (particularly in relation to sports), the meaning of sports activities for the individual well-being, the presence or absence of comorbidities and of neurological sequela, neurovascular findings, and whether there are signs of an underlying connective tissue alteration. Major limitations and several forms of bias are acknowledged. Still, in the absence of any better data, the summarized observations and considerations might help clinicians in advising and counseling patients with CeAD in clinical practice.

## Introduction

Cervical artery dissection (CeAD) is a major cause of stroke in younger adults (1, 2). Mostly, CeAD occurs in previously healthy individuals who usually lack atherosclerotic risk factors, with the exception of hypertension (3). Just over 40% of CeAD patients report cervical trauma, a potential mechanical trigger event (4). Sports-related trauma accounts for an important subset of such trauma (4). The association between sports and stroke attributable to CeAD (5–7), e.g., “golfer's stroke” (8, 9), indicates the possibility of harm of the cervical brain supplying arteries by active sports. On the other hand, low physical activity is an important risk factor for stroke in general and predicts unfavorable functional outcome after stroke (10, 11). The positive impact of physical activity and sports on vascular health has led to the slogan “exercise is vascular medicine”(12).

Patients with CeAD associated with sport are particularly concerned about the possible harm of sports they have practiced. They seek advice on the safety of resuming sport activities after CeAD, on the appropriate timing, and about which form and intensity of sport. CeAD patients need informed counseling, but the scarcity of studies addressing the role of sports in CeAD poses a major challenge. Current stroke guidelines do not comment on whether, how, or when sports should be resumed (13, 14). Thus, summarizing the current knowledge about CeAD and sports and to provide an informed, comprehensive perspective has substantial impact for counseling CeAD patients.

For the purposes of this article, we consider sport as practicing any of the sport disciplines represented by Sport Accord (15), an umbrella organization connecting all Olympic and non-Olympic sport federations. In line with the European Union's definition of sports (16), the European Sports Charter (17), and a recent case series (7), we did not include sport disciplines primarily involving cognitive activities such as chess, or motor sports, or electronic sports.

### Pathophysiological Considerations

**Figure 1:**
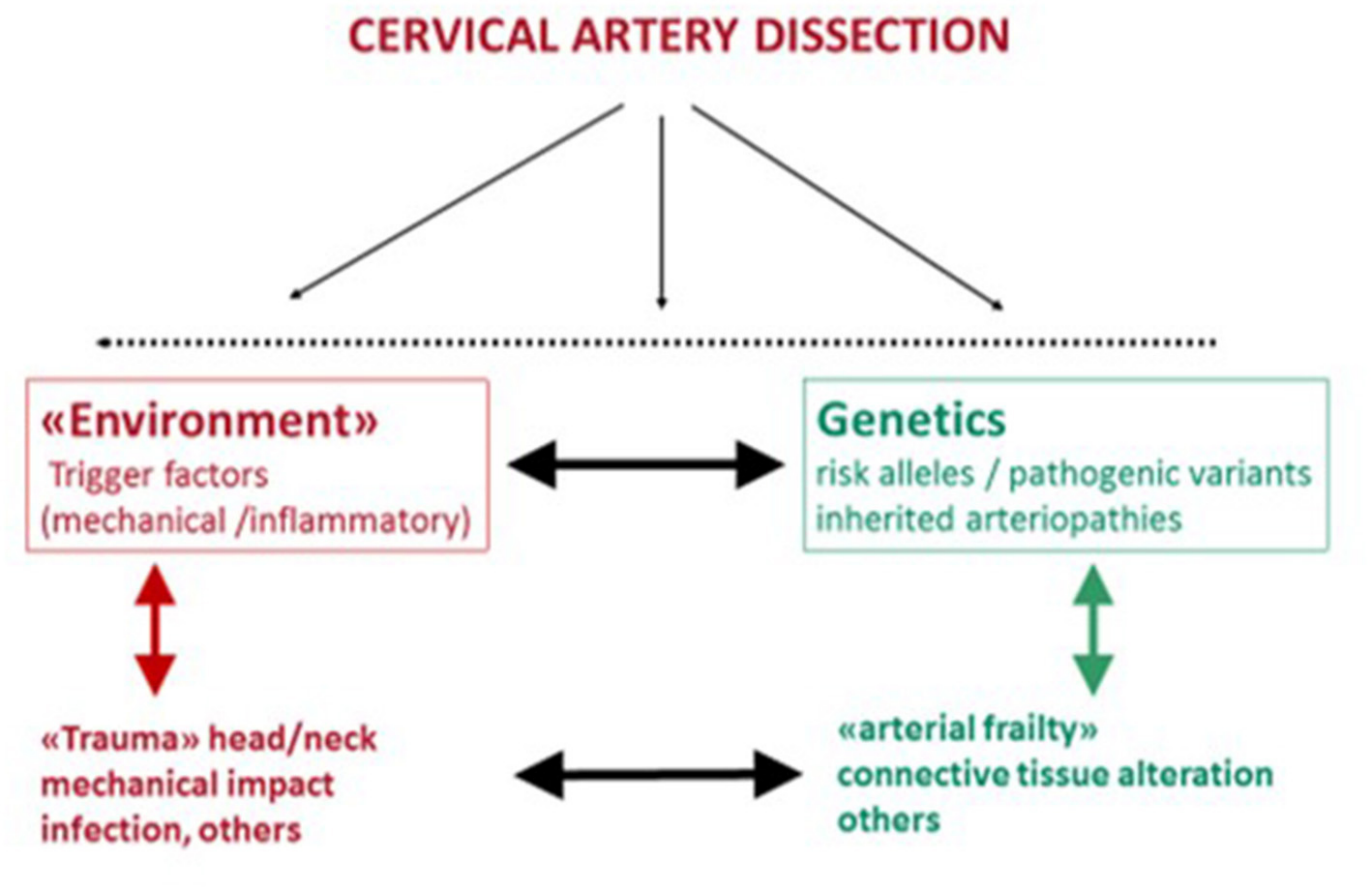
Etiology of CeAD as a multifactorial disease with environmental and genetic risk determinants that probably interact.

Although the pathophysiology is unclear (18–20), CeAD is assumed to be a multifactorial disease, with an interaction between environmental determinants and genetic factors (Figure 1). In skin biopsies, approximately half of CeAD patients show mild morphologic alterations of their connective tissue ultrastructure (21, 22). As the skin can be considered as a “window of heritable disorders of connective tissue” (23), this observation suggests that mild inherited connective tissue aberrations may weaken arterial connective tissue (“arterial frailty”). Indeed, known inherited connective tissue disorders such as Marfan syndrome, Ehlers-Danlos syndrome, or Loeys-Dietz syndrome predispose to CeAD, but are rare [<1% (24)]. However, mild clinical signs of a connective tissue weakness, including slight joint hypermobility, thin and translucent skin, or easy bruising, are common and associated with CeAD (25, 26).

The Cervical Artery Dissection and Ischemic Stroke Patients (CADISP) consortium aimed at exploring genetic (27), environmental (3, 4), and treatment (28) aspects in more detail. In CADISP, consecutive patients diagnosed with CeAD and patients with ischemic strokes without CeAD were enrolled at neurology departments in eight countries (27). Diagnosis of CeAD was based on widely accepted diagnostic criteria, mostly magnetic resonance imaging findings (3, 4, 28), and standardized questionnaires were used to assess clinical data in all participants. More CeAD patients than controls harbored structural genetic variants affecting cardiovascular system development, suggesting the presence of an underlying cardiovascular vulnerability (29, 30). In addition, patients with CeAD, particularly in those with familial CeAD, had rare, pathogenic variants in genes associated with connective tissue disorders (31, 32). Moreover, a Genome-Wide Association Study revealed association with a common variant in the PHACTR1/EDN locus (33). Interestingly, independent studies showed association of the same allele of this PHACTR1/EDN1 polymorphism with a variety of other vascular phenotypes, including fibromuscular dysplasia, spontaneous coronary artery dissection, and migraine (34). In the CADISP study population, there was no interaction between the PHACTR1/EDN1 risk allele and reported trauma (33). However, the study was not powered to detect such an interaction.

Mechanical triggers are of interest in the context of sports. The CADISP consortium sought to evaluate the clinical importance of trauma and other mechanical events in CeAD by analyzing characteristics of trauma in the neck and head region 1 month prior to the event. Patients, with ischemic stroke attributable to causes other than CeAD and healthy individuals, both sex- and age-matched to the CeAD patients, served as comparators. Approximately 40% of the CeAD patients reported any kind of recent head or neck trauma in the month prior to symptom onset (4), as compared to 10% of the patients with ischemic stroke attributable to a cause other than CeAD and 20% of the healthy controls (4). More than 90% of the trauma events recalled by the CeAD patients were trivial and so mild that the individuals did not seek for medical care or advice. As a causal relationship with the CeAD is either questionable or unclear, the term *mechanical trigger event* is preferred.

### Sports-Related CeAD

In a so far unpublished refinement of the aforementioned analysis, sports were self-reported trigger factors in 61 of 966 CeAD patients (6.3%), compared to five of 651 patients (0.8%) with ischemic stroke attributable to a cause other than CeAD. The same kind of mechanical events or trauma while practicing sports has also been reported by five of 280 healthy controls (1.8%) (*p* < 0.001). However, the main limitation of this *post hoc* analysis is that the CADISP questionnaire did not explicitly include questions on sports activities. Moreover, the desire of causality may have influenced the self-reported information about triggers in CeAD patients rather than in the comparison groups, indicating the possibility of a recall bias.

A joint evaluation of the stroke registries of the universities of Lisbon and Porto reported solely 10 patients with stroke occurring in temporal association with sport activity over a 2-year observation period (35). In six of these 10 patients, strokes were attributable to CeAD. These data indicate that the prevalence of sports-related stroke and CeAD is likely to be very low.

The distinction between spontaneous vs. sport-associated CeAD may appear attractive at the first glance, but is often an oversimplification and not helpful in clinical practice (36), as mechanical factors may be a trigger even if there is an underlying genetic predisposition.

The necessity to consider environmental as well as genetic factors—even if a mechanical trigger seems obvious—is illustrated by the case of a patient with osteogenesis imperfecta and a pathogenic *COL1A1* mutation who developed multivessel CeAD after scuba diving (37).

It is unclear why patients with a CeAD event during a regular sports activity, e.g., swinging a golf club while playing golf, have the CeAD “this time” and not the hundreds of times they played golf before. Furthermore, CeAD can occur during the wide variety of daily life situations other than sport activities. This includes CeAD after delivery, sexual intercourse, dancing, or playing music, as a consequence of a roller-coaster ride, after a visit to a dentist or the hairdresser, or even after a neurological examination. The onset of CeAD during daily activities or common sports (such as running, walking bicycling) does not necessarily prove causality, but may be pure coincidence.

The following variables might be helpful to get an idea on whether the CeAD might—at least in part—be caused or triggered by a sports activity: first, the time between sports activity and symptom onset (7); second, the occurrence of blunt forces affecting the neck, the mastoid region, or the head (5); third, specific dynamic or static characteristics of the involved strain during sports (7); fourth, whether an accident with involvement of the head and neck happened in the sense of as a sudden and unwanted event disrupting regular sports activities. As a rule of thumb—for clinical purposes rather than in a medicolegal context—arguments in favor of a causal relationship include the following:

- A short delay between sports activity and onset of CeAD symptoms, in particular, if they occurred immediately after or already during sport activity. According to a recent review (7), the mean delay was 4.8 days. In this report, the onset of symptoms occurred during sport activity or immediately after the reported sport activity, in approximately half of the patients. However, in the other half, the delay was much longer and lasted up to 6 months (7).
- A sports-related direct −60% in a recent review (5)—or indirect blunt trauma of the neck or head preceding the onset of CeAD symptoms.
- When sport activity involved jerky, rapid movements; abrupt rotation; triggered reflexive reactions; long-lasting hyperextension; or rapid flexion–extension of the neck or very high blood pressure values and when the neck was rotated with or without extension (5, 7). Whereas in some sport activities direct blunt trauma (e.g., combat-sports) or indirect trauma (e.g., golf) or increased intrathoracic pressure with high blood pressure (e.g., weight lifting) may be predominant, a combination of the aforementioned components may be present in other sport activities [e.g., swimming, climbing, or bicycling (7)].

#### Different Types of Sport Activities

A review of 190 suspected sport-associated CeAD cases (7) covered such different sport activities as combat sports (judo, wrestling, kickboxing), weightlifting, running/jogging, scuba diving, swimming, skiing, yoga, golf, or horse riding. Patients were young (mean age = 35 years), and 26% were women. The broad range of sports associated with CeAD suggests differential of risk mechanisms between different types of sports. However, publication bias is likely to be present. Several categories have been proposed: contact vs. noncontact sports (5), competitive vs. recreational sports (38), and dynamic vs. static efforts (39).

Types of sports related to CeAD differ with advancing age and sex. Whereas, football, basketball, and combat sports are more frequently reported in younger patients, golf was predominantly reported in older patients (7). Moreover, whereas running was the most frequent type of sports activity in women with sports-related CeAD, for men, combat-sports and golf were more common in men (7). These differences most likely reflect age- and sex-related preferences in sports activities in the general population rather than a sex-, gender-, or age-specific harm of different sport activities.

**Figure 2:**
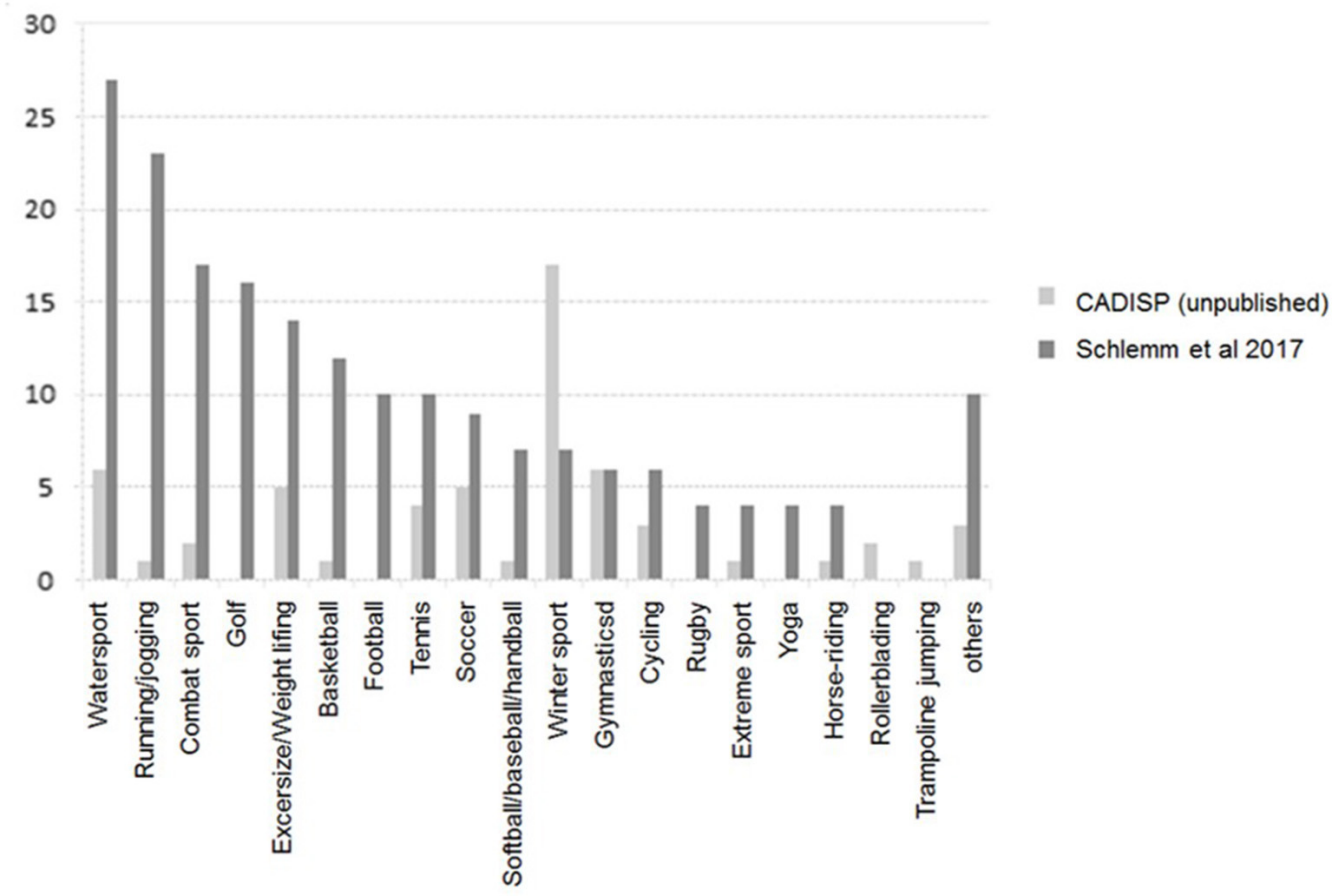
Frequency of CeAD-associated sport types in the review of case series by Schlemm et al. (7) and in the CADISP study population.

The frequency of types of sport activities reported in relation to CeAD differed between the aforementioned review (7) and the CADISP cohort. In the latter, winter sport activities predominated (Figure 2). In CADISP, several patients were recruited in Finland and Switzerland, where winter sport activities are popular. Thus, the discrepancy with the distribution in Schlemm et al. may simply reflect differences in sport practice pattern across different countries or regions. The popularity of doing active sports in general and of practicing particular types of sports differs across countries (40). Therefore, the association of CeAD events with particular sport types is likely to be affected also by country.

Mortality rates of 17 and 7% in reviews summarizing sports-associated CeAD events (5, 7) exceed those of hospital-based registries of CeAD patients (1, 2). Although this difference may suggest a poorer prognosis of sports-related CeAD than CeAD in general, the risk of an underlying recruitment bias (i.e., participation in CADISP required informed consent), as well as a publication bias, is likely to be present.

## Discussion

In analogy with findings in aortic dissection, excessively high blood pressure values are assumingly as harmful in CeAD as in aortic dissections. CeADs as well as aortic dissections have been reported related to heavy weight lifting (7, 39). Furthermore, unfavorable blood pressure effect on the arterial wall has been observed in a model of CeAD (41). Peak blood pressures approaching 300 mm Hg were observed during severe weight lifting (39) with even more dramatic values in occasional individuals (42). CeAD patients carrying out this kind of sport should refrain from lifting heavy weights, as it is for patients who had had an aortic dissection: “The European Society of Cardiology highlights that in patients with (aortic) dissection, competitive sports and isometric heavy weightlifting should be discouraged” (43). As sport activity involving jerky, rapid movements; abrupt rotation; long-lasting hyperextension; or rapid flexion–extension of the neck may trigger CeAD, such sports as well as those with a high risk for accidents should be discouraged in patients with CeAD (5, 7) for at least 6 months.

Recommendations about sports in patients with inherited arteriopathies (44) may be of interest for CeAD patients. Patients with Marfan syndrome and aortic dissections (44, 45) should refrain from sports activities involving collision including a high risk for accidents (e.g., falls) and heavy contact, avoid isometric exercise, and only participate in activities with low-intensity, low-dynamic, and low-static components (46). For Marfan syndrome patients with previous aortic root reconstruction, the guidelines prohibit any competitive sports participation classified more than low in intensity (47). As <1% of CeAD patients do have Marfan syndrome, these recommendations may be too strict for most CeAD patients.

Disease-causing pathogenic mutations were reported in only a minority of CeAD patients. In patients with mutations affecting the arterial connective tissue integrity (30, 32), recommendations as for patients with Marfan syndrome (44, 45) seem justified. For the majority of patients, however, genetics remains without relevant clinical impact regarding sports activities. Variation in the LRP1 and the PHRACT1/EDN loci was associated with CeAD (33), but an interaction with mechanical trigger factors was not shown, although the study was not powered to show or exclude such an association. It has been a matter of speculation whether patients with mild clinical signs of a connective tissue alteration are more prone to hyperextension of the neck (25). However, as far as we know, an increased risk of CeAD during sport activity in patients with connective tissue signs has not been reported.

Whether or when to restart sport activities is another frequently asked question by patients. In absence of clear evidence, this question requires—in our opinion—an individual decision taking into account the circumstances of the occurrences of the CeAD in the individual patient (particularly in relation to sports), the meaning of sports for the individual well-being, the presence or absence of comorbidities or of neurological sequela, neurovascular findings, and whether there are signs of an underlying connective tissue alteration. Further, recurrence of CeAD is infrequent, and clusters within the first weeks to 3 months after the index CeAD (47, 48) and strokes attributable to CeAD do occur (or reoccur) with a preference for the first 2 weeks after diagnosis (49). Serial ultrasound examinations in CeAD patients identified the occurrence of new arterial findings, of which several were associated with clinical symptoms in particular in the first 4 weeks. These findings included five recanalizations of initially occluded dissected arteries (50). Although a relationship with sports activities was not reported, these observations suggest an unstable neurovascular situation during which sports activities might be harmful. Thus, it seems recommendable to refrain from any kind of sports activities—which includes also light activities—for a period of at least 1 month, which can be extended in case of an unfavorable clinical or neurovascular course. Thereafter, one can probably recommend gradual resumption of sports activities to most CeAD patients for the following reasons. In general, sports activity is a well-established protective factor in prevention of ischemic stroke, mainly due to its influence on common vascular risk factors, such as blood pressure (51). Furthermore, psychological sequelae are common after stroke and in particular post-CeAD (52, 53). Such symptoms can be alleviated by graded activity training (54) and sports as it has been shown to work for fatigue (55, 56) and depression (57). We assume that such benefits of sports are valid also for patients after CeAD, even though studies addressing the impact of sports on the long-term outcome after CeAD are missing, although CeAD patients differ from other stroke patients in many aspects including risk factors and socioeconomic levels (3, 58, 59).

We usually recommend to start with sport activities at low intensity—preferably with types of endurance sports—if patients have recovered neurologically and are in stable neurological and normalized hemodynamic condition for at least 1 to 3 months. Medication should be taken into account; e.g., decision for temporary oral anticoagulation may influence the time point to resume sports with a higher risk of injury. If a decision has been made in favor of oral anticoagulation, one may want to await its ending to reduce the likelihood of severe bleeding complications.

CeAD patients with inherited connective tissue disorders, such as Marfan syndrome, Ehlers-Danlos syndrome, or Loeys-Dietz syndrome, should permanently avoid combat sports because of the risk of collision and heavy contact, avoid strong isometric exercise, and preferably participate in sports activities with low-intensity, low-dynamic, and low-static components—in conclusion by analogy with aortic dissection. For patients without underlying inherited connective tissue disease, a gradual increase in the sports activity is recommended after 3 months of abstaining sports activity with strong exercise.

After 6 to 12 months, CeAD patients may consider to restart pace and pattern of the sports activities they were used to prior to the CeAD with the following general recommendation. Patients should avoid jerky head–neck movements or abrupt rotation, long-lasting hyperextension, or rapid flexion–extension of the neck, as much as possible, in particular at the beginning of the resuming phase. Furthermore, they should minimize the likelihood as well as the impact of sport-associated trauma of the neck or head by acting carefully and by carrying protective devices including helmets not only for sports for which such is required (e.g., ice hockey, American football) but also for cycling, soccer (goalkeeper), or horseback riding. However, helmets, unfortunately, usually do not protect the neck and may even enhance a head tilt in case of some blunt or deceleration trauma. Furthermore, awareness of own physical limits and strict avoidance of practicing sports while overtired are recommendable.

## Limitations

The available data on sport-associated CeAD came from single-case studies or small case series or were retrospective analyses from registries. Studies were highly heterogeneous, included different types of sports, analyzed professional athletes sports or leisure activities, and regular sport activity with or without a trauma. Systematic, prospective studies are missing. Several forms of bias have to be taken into account. This study took into account data from a recent review (7) and from a large registry (4). However, there are important differences between these datasets: Schlemm et al. collected and analyzed a large number of case reports and small case series of CeAD events associated with various sport events, including fatal cases. No fatal cases were reported by the CADISP study. All patients gave informed consent to participate in the CADISP study. For data collection, a uniform structured questionnaire was used with detailed questions on clinical presentation and risk factors, including mechanical triggers. However, there were no structured questions on physical activity and sport activity. Detailed information on the type of mechanical trigger events, including sports, was added as unstructured comments by most but not all CADISP investigators.

Data on sports activity after CeAD are virtually absent and do not permit conclusions balancing risks and benefits of sports activity in patients after CeAD. Thus, the aforementioned recommendations are therefore nothing more than opinions of experienced clinicians, based on personal experiences, case reports, tradition, clinical reasoning, and common sense, and can be adapted to individual situations.

In conclusion, CeAD seems to not frequently occur in the context of sport activities. Moreover, practicing sports as the cause of CeAD seems uncommon. However, the level of evidence is low, and the likelihood of bias is high. Therefore, we urge to cautiously interpret our conclusions. Data about resuming sports after CeAD are virtually absent. Restarting sports after temporarily stopping sports activity in a gradually increasing pace and in an individually tailored manner lacks evidence but reflects clinical reasoning. This article is not meant to address any mediolegal aspects. Still, in the absence of any better or more robust data, the reported observational data and considerations might help clinicians in advising and counseling patients with CeAD in clinical practice.

## Data Availability Statement

The data analyzed in this study is subject to the following licenses/restrictions: Multicentre international collaboration with local regulatory restrictions to make data publicly available. Requests to access these datasets should be directed to stefan.engelter@usb.ch.

## Author Contributions

SE, TT, and PL: study design, drafting and reviewing the manuscript, and review of literature. CT, and CG-G: drafting and reviewing of manuscript and extraction and review of literature. TB, MH, BW, SD, AP, DL, and CN: critical review of manuscript. All authors contributed to the article and approved the submitted version.

## Conflict of Interest

The authors declare that the research was conducted in the absence of any commercial or financial relationships that could be construed as a potential conflict of interest.
